# Winter microbial community structure and methane-cycling potential in constructed agricultural wetlands across regions and microhabitats

**DOI:** 10.1093/femsec/fiaf086

**Published:** 2025-09-03

**Authors:** Tong Liu, Klara Li Yngve, Martyn Futter, Mike Peacock, John Strand, Stefan Bertilsson, Pia Geranmayeh

**Affiliations:** Department of Aquatic Sciences and Assessment, Swedish University of Agricultural Sciences, Uppsala, 75007, Sweden; Department of Aquatic Sciences and Assessment, Swedish University of Agricultural Sciences, Uppsala, 75007, Sweden; Department of Aquatic Sciences and Assessment, Swedish University of Agricultural Sciences, Uppsala, 75007, Sweden; Department of Aquatic Sciences and Assessment, Swedish University of Agricultural Sciences, Uppsala, 75007, Sweden; Department of Geography and Planning, School of Environmental Sciences, University of Liverpool, Liverpool, L697ZT, United Kingdom; Hushållningssällskapet Halland, Eldsberga, 30596, Sweden; Department of Aquatic Sciences and Assessment, Swedish University of Agricultural Sciences, Uppsala, 75007, Sweden; Science for Life Laboratory, Uppsala, 75108, Sweden; Department of Aquatic Sciences and Assessment, Swedish University of Agricultural Sciences, Uppsala, 75007, Sweden

**Keywords:** 16S rRNA, constructed wetlands, emergent vegetation, methane, methanotrophs, microbial community, winter

## Abstract

Constructed wetlands are widely used to reduce nutrient loading to downstream waters, but they can also emit methane, a potent greenhouse gas. This trade-off between water quality benefits and climate impacts is driven by microbial processes that remain poorly understood in winter. We examined microbial community composition and methane-cycling potential in surface water samples from constructed wetlands in two agricultural regions of Sweden during the winter season, focusing on the effects of emergent vegetation and environmental conditions. Western wetlands, characterized by higher total nitrogen and dissolved oxygen, exhibited significantly greater microbial diversity and more complex co-occurrence networks than eastern wetlands. At the phylum level, Actinobacteriota and Firmicutes were more abundant in the west, while Bacteroidota dominated the east. The effects of emergent vegetation were region-specific: in the west, vegetated zones supported higher diversity and enrichment of plant-associated taxa. Several taxa affiliated with methanotrophs showed higher relative abundance in vegetated zones of the western wetlands, suggesting vegetation may enhance methane oxidation potential in surface waters, even though methane concentrations were similar. Overall, winter microbial networks remained structured, emphasizing the need for integrated microbial and biogeochemical studies to guide wetland design features, such as vegetation and nutrient regimes, that support both methane mitigation and nutrient retention in cold-climate agricultural landscapes.

## Teaser

This study reveals how vegetation and regional context shape microbial networks and methane-cycling communities during winter in constructed wetlands, offering new insights into cold-season C1 metabolism. Created in BioRender. Liu, T. (2025) https://BioRender.com/h6morz2.

## Introduction

Constructed wetlands emulate the functions of their natural counterparts to provide crucial ecosystem services, including nutrient retention and biodiversity conservation. As such, they can play a pivotal role in mitigating environmental impacts of agriculture on downstream ecosystems (Vymazal 2011, Mitsch et al. 2013, Spieles 2022). In many locations where diffuse agricultural nutrient leaching contributes to eutrophication, constructed wetlands are integral to environmental management strategies (Land et al. 2016, Geranmayeh et al. 2018, Ioannidou and Stefanakis 2020). However, the same conditions that support nutrient retention, saturated soils and anaerobic zones, also promote methane (CH_4_) production. This creates a trade-off: constructed wetlands reduce nutrient loads to downstream waters, but can also emit methane under anaerobic conditions, raising concerns about their net climate impact (Yin et al. 2023).

The CH_4_ cycle within wetlands is a complex interplay of microbial processes that are heavily influenced by plant community and seasonal temperature variations (Xu et al. 2019). CH_4_ production is mediated by methanogenic archaea living in anaerobic conditions. The process is linked to the broader microbial community, including bacteria that decompose complex organic matter into simpler molecules that methanogens can utilize (Conrad 2009). Methanotrophs can subsequently oxidize CH_4_ to carbon dioxide in the presence of oxygen. Rates of CH_4_ production and consumption are contingent on microbial community structure, which can either facilitate or compete for resources necessary for CH_4_ oxidation (Knief 2015). The role of aquatic vegetation in controlling these microbially-mediated processes is complex. Plants can enhance CH_4_ emissions by releasing easily degradable organic compounds (e.g. root exudates) that serve as substrates for methanogenesis (Ström et al. 2012) and create conduits for evasion of CH_4_ to the atmosphere through their aerenchymatous tissues (Xu et al. 2019). During the dormant season, the breakdown of dead plant tissue may also contribute labile carbon that supports methane production (Ueyama et al. 2023). Simultaneously, aquatic plants can also bring oxygen into the rhizosphere, thereby promoting CH_4_ oxidation (Roura-Carol and Freeman 1999, Turner et al. 2020). The distinction between vegetated and open water areas within constructed wetlands, therefore, provides a critical framework for examining microbial community interactions that drive CH_4_ dynamics.

Seasonality further complicates these dynamics. CH_4_ emissions from constructed wetlands are generally highest during summer (Johansson et al. 2004, Pangala et al. 2010), reflecting not only the positive temperature dependence of methanogenesis (Yvon-Durocher et al. 2014), but also increased plant activity during the growing season. While the majority of the literature suggest that CH_4_ fluxes are greatest during warmer seasons, winter emissions are often underreported, possibly due to a lack of sampling during this period. This could result in significant emissions being overlooked even if they may not exceed summer levels. In fact, wetland CH_4_ emissions during the cold (no growth) season can contribute 13%–47% of annual emissions (Treat et al. 2018). These emissions likely reflect a combination of factors, including ongoing methanogenesis at depth, where temperatures may be more stable, and reduced, but not fully inhibited, methanotrophic activity (Zimov et al. 1997). This aligns with temperature sensitivity estimates, which show that methanogenesis is typically more inhibited by low temperatures (Q₁₀ often >3), yet can persist under favorable anaerobic conditions. In contrast, cold-adapted methanotrophs may remain active near freezing, potentially limiting CH_4_ accumulation in oxygenated surface layers (Zhu et al. 2014). Conversely, the cold also impacts plant metabolism and methanogenesis, which could lead to reduced methane production. Furthermore, many studies show an accumulation of CH_4_ under winter ice that is released as a large pulse during spring thaw (Johnson et al. 2022). However, CH_4_ that is trapped beneath the ice is also exposed to methanotrophs, potentially enabling a more far-reaching CH_4_ oxidation, and this effect is expected to be greater in phosphorus-rich waterbodies (Sawakuchi et al. 2021). It is evident that our understanding of winter CH_4_ dynamics, as well as the role of plants during the dormant season, has significant gaps. Further information is essential for informing the design and management of constructed wetlands, such as optimizing vegetation composition, hydrological regimes, and oxygen delivery, to support microbial communities that enhance methane mitigation while maintaining nutrient removal efficiency (Wang et al. 2016, Yu et al. 2023).

In this study, we test the hypothesis that emergent vegetation influences microbial community and methane-cycling processes in wetlands. In doing so, we bridge a gap in understanding to inform about the CH_4_ cycle in constructed wetlands in agricultural landscapes during the winter season, with emphasis on areas with and without emergent vegetation and complex interactions within the microbial communities.

## Methods and materials

### Sites description and sampling

Water samples were collected from 34 constructed wetlands in agricultural areas of two contrasting regions of Sweden: Halland (west, 13 wetlands) and Mälardalen (east, 21 wetlands) (Table S1). Halland (56°44′ N 12°58′ E) has an oceanic climate and while the region is characterized by sandy soils, the constructed wetlands are typically built on clay subsoils to retain water, often overlain by sand. Mälardalen (59°47′ N 17°30′ E) has a humid continental climate and predominantly clay soils. Wetlands in the eastern region were on average 6 years old, whereas those in the western region averaged 18 years since construction or maintenance (Table S1). Sampling took place between January 31 and February 11, 2022, at two sites within each wetland: one open water (lacking emergent vegetation) site and one site with emergent vegetation, selected to represent typical microhabitats within the wetland. The vegetated site was generally close to the edge, while the unvegetated site was positioned 2–5 m from the shore where possible; in some cases, site positions were reversed depending on vegetation distribution. At each site, water depth was measured with a folding ruler, and integrated water-column samples were collected. For depths >0.3 m, a 4-l Ruttner sampler was used; for shallower sites, a 1-l handheld bailer was employed, taking care to avoid sediment disturbance. Ice thickness (10–50 cm at most eastern sites) was recorded but excluded from water depth measurements. In the western wetlands, where most sites were ice-free, samples were collected using a stand-up paddleboard (STRAND et al. 2023). In the eastern wetlands, most sites were ice-covered and accessed by walking on the ice and using an ice drill. At two eastern sites, E03 (ice-free) and E16 (with thin, unsafe ice), the paddleboard was used to safely access open water for sampling (Yngve 2022).

### Water chemistry and CH_4_ sampling

At each site, water depth was measured with a folding ruler. Surface water temperature, pH, dissolved oxygen, and electrical conductivity were determined using a Hanna HI 9829 Multimeter. Chlorophyll *a* concentration was measured with a FluoroSense™ handheld fluorometer (Yngve 2022). Surface water samples were collected in Nalgene bottles and analyzed at the SWEDAC-accredited Geochemical Laboratory at the Swedish University of Agricultural Sciences (SLU) for concentrations of total organic carbon (TOC), total nitrogen (TN), ammonium (NH_4_), total phosphorus (TP), and suspended solids. All methods and analytical techniques are documented thoroughly online (SLU 2024).

Surface water CH_4_ concentrations were measured at each sampling site using the headspace technique (Hope et al. 2004). Thirty milliliter of surface water from the wetland was drawn into a 60-ml syringe, along with 30 ml of atmospheric air collected ∼1 m above the water surface. The syringe was vigorously shaken for one minute and then 15 ml of headspace air was injected into a 12-ml glass Exetainer vial. Gas samples were analyzed with a Perkin Elmer Clarus 500 gas chromatograph and CH_4_ concentrations were calculated using the solubility function of Wiesenburg and Guinasso (1979) (Wiesenburg and Guinasso 1979) and adjusting for water temperature, atmospheric pressure, and ambient air concentration.

### DNA extraction from water samples

Integrated water samples were collected to capture the entire water column, using a Ruttner sampler for depths over 0.3 m and a handheld plastic bailer for more shallow sites. Once collected, water was pooled in a 10-l bucket and filtered through 0.2 µm Sterivex™ filters using a 60 ml syringe, with filtration volumes adjusted as needed to avoid clogging. Samples were stored in the dark at +4°C and filtered within 7 days of collection. Filters were then frozen at −18°C until DNA extraction.

The plastic cylinder surrounding each Sterivex filter was opened with a pair of pliers before DNA extraction. Filters were then carefully removed using a disposable razorblade and tweezers, and subsequently cut into halves or thirds. To ensure a contamination-free environment, all tools and the workspace were thoroughly cleansed with 70% ethanol before and after handling each sample. Filters were placed into sterile 5-ml plastic tubes (Sarstedt AG & Co. KG, Nümbrecht, Germany) for immediate DNA extraction.

The DNA extraction for each site (68 samples in total) was done with the DNeasy^®^ PowerSoil^®^ Pro Kit (QIAGEN), following the kit's protocol with modifications to the initial steps. Specifically, the PowerBead Pro tubes' microbeads, provided in the kit, were transferred into the tubes containing the filters along with 800 µl of Solution CD1. These tubes were then vortexed for 5 min at 2.60 m/s using a Fisherbrand™ Bead Mill 24 Homogenizer. Subsequently, the supernatant was transferred into clean 2-ml microcentrifuge tubes, adhering strictly to the remaining steps of the protocol without deviations. Finally, 6 µl of the extracted DNA from each sample was diluted with 54 µl of DNase/RNase-free water and stored at −18°C for subsequent methanotroph quantification and microbial community analysis. DNA concentration in extracts was quantified by using Qubit 1X dsDNA Broad Range (BR) Assay Kits with a Qubit 3.0 Fluorometer (Invitrogen, Thermo Fisher Scientific, Waltham, MA, USA).

### Microbial community analysis

The 16S rRNA gene amplicon library for Illumina sequencing was prepared by polymerase chain reaction (PCR) using the primer pair 341F(CCTACGGGNGGCWGCAG) and 805NR(GACTACNVGGGTATCTAATCC) for the microbial community (Herlemann et al. 2011). This primer set showed 95% and 89.8% coverage of bacteria and archaea, respectively, in the SILVA SSU r138.2 (RefNR) database (mismatches < 2, tested on 2025–02–24), suggesting that genes from both bacterial and archaeal domains would be adequately amplified (Table S2). The detailed PCR procedure and the sequence library preparation steps have been described elsewhere (Sinclair et al. 2015). To minimize the impacts of spurious amplification in the first amplification cycles, the initial PCR was conducted in duplicate for each sample, with the products combined for a subsequent PCR where molecular barcodes were introduced. Next-generation amplicon sequencing was performed in-house using Illumina MiSeq technology with Reagent Kit v3. The raw data were analyzed with open-source bioinformatics pipeline DADA2 (version 1.16) as implemented in R (version 4.0.2) (Callahan et al. 2016). Forward and reverse primers were trimmed before merging paired reads, and the resulting sequences were cut to lengths of 279 and 243 bp, respectively, with the quality threshold of maxEE = (3, 3) and truncQ = 2, according to in silico calculation by FIGARO (Weinstein et al. 2019). Taxonomic profiles were assigned based on amplicon sequence variants (ASVs) using the rRNA database SILVA, release 138.1 (Quast et al. 2013). The sequencing raw data can be accessed via the National Centre for Biotechnology Information (NCBI) database (BioSample accession SAMN46919064 under BioProject PRJNA1226119).

### Statistical analyses

ASVs classified as Chloroplast were removed from the dataset prior to all downstream statistical analyses and figure generation. Permutational multivariate analysis of variance (PERMANOVA; adonis2, R package vegan, version 2.6–4) was employed to assess differences in microbial community composition. Bray–Curtis dissimilarities were used as input, and models included environmental variables and categorical groupings such as Region and Vegetation type. ASVs were rarefied to correspond with the minimum read count observed across the samples (5 640 ASVs). Non-metric multidimensional scaling was utilized for visualizing the overall dissimilarity among microbial communities in the samples (vegan, version 2.6–4, permutations = 999). Group-level ellipses (95% confidence intervals) were generated using the stat_ellipse() function in ggplot2, based on the multivariate normal distribution of samples grouped by Region. For analyzing and visualizing differences in microbial community compositions at the genus level between wetland locations and emergent vegetation presence/absence, ANOVA and Linear Discriminant Analysis (LDA) were conducted using the Phyloseq (version 1.44.0) (McMurdie and Holmes 2013) and MicrobiotaProcess (version 1.12.3) (Xu et al. 2023) packages. The LDA analysis consisted of two stages. In the first stage, the Kruskal–Wallis test was applied to identify statistically significant differences in microbial taxa across subset groups (*P* ≤ 0.05). In the second stage, pairwise differences were evaluated using the Wilcoxon test, with the same significance threshold (*P* ≤ 0.05). Co-occurrence network analysis was performed at the genus level, based on genera representing >1% of the total sequence reads. Spearman's rank correlation coefficients (ρ) were calculated between genus-level relative abundance profiles, using RCy3 and the igraph package in R. *P*-values were adjusted using the Benjamini–Hochberg correction to control the false discovery rate (Haynes 2013). Only genus pairs with strong and significant correlations (|*ρ*| > 0.5 and adjusted *P* < 0.05) were retained for network construction. Network structure indices such as degree, betweenness, and closeness centrality were calculated to assess network architecture and pinpoint keystone microorganisms (Berry and Widder 2014). The resulting co-occurrence network was depicted in Cytoscape (version 3.7.2).

## Results

### Wetland water chemistry

**Figure 1.**
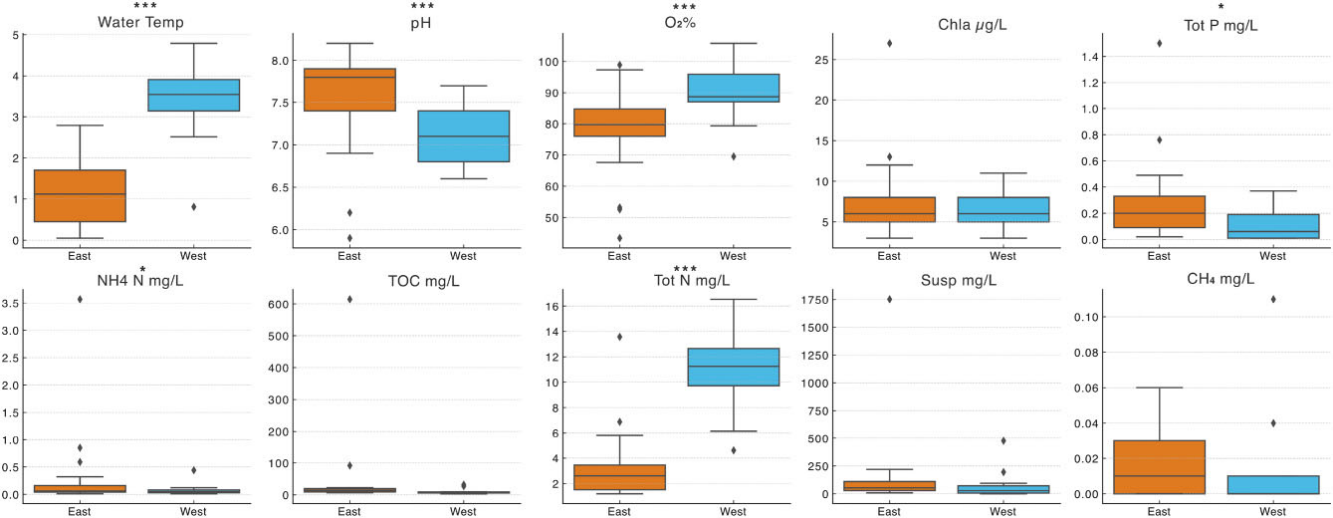
Comparison of environmental variables between eastern and western wetlands during winter. Boxplots show distributions of water temperature (°C), pH, dissolved oxygen (O₂%), chlorophyll-a (Chla), total phosphorus (Tot P), ammonium (NH_4_⁺), total organic carbon (TOC), total nitrogen (Tot N), suspended solids (Susp), and methane (CH_4_) concentrations. Asterisks indicate statistically significant differences between regions based on Mann–Whitney U tests (*P < 0.05, **P < 0.01, ***P < 0.001).

Wetland water chemistry differed substantially between the two regions (Fig. 1, Table S3). Compared to eastern wetlands (Mälardalen), western wetlands (Halland) had significantly higher total nitrogen (TN: 10.84 ± 0.59 vs. 3.33 ± 0.21 mg l^−1^) and dissolved oxygen (O₂) (89.4 ± 1.8% in the west vs. 77.9 ± 2.1% in the east), while eastern wetlands had significantly higher total phosphorus (TP: 0.29 ± 0.05 vs. 0.13 ± 0.04 mg l^−1^) and ammonium (NH_4_⁺: 0.32 ± 0.12 vs. 0.06 ± 0.01 mg N l^−1^). Mean pH was higher in the east (7.55 ± 0.09) than in the west (7.11 ± 0.07). Chlorophyll-a concentrations were similar (7.8 ± 0.8 µg l^−1^ in the east vs. 6.6 ± 0.5 µg l^−1^ in the west). The east also had higher TOC (45.9 ± 20.1 vs. 9.7 ± 1.7 mg l^−1^) and suspended solids (150 ± 57 vs. 74 ± 26 mg l^−1^). Methane (CH_4_) concentrations ranged from below detection to 0.11 mg l^−1^, with slightly higher mean levels in the east (0.017 ± 0.003 mg l^−1^) than in the west (0.015 ± 0.006 mg l^−1^), though not statistically significant (*P* > 0.05). No significant differences in CH_4_ concentrations were observed between samples collected from emergent vegetation and open water areas, either across all wetlands or within each region (*P* > 0.05).

### Overall microbial community distribution

**Figure 2.**
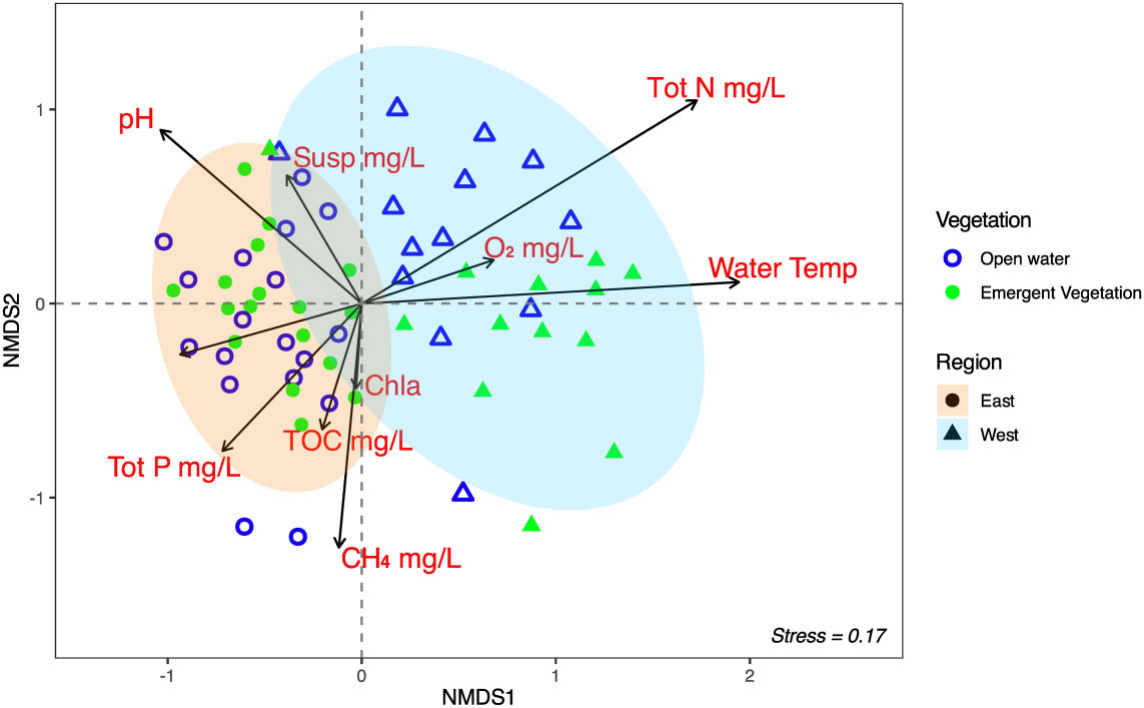
Non-metric multidimensional scaling ordination based on relative abundance of amplicon sequence variants of microbial 16S rRNA amplicons. Dots shaped according to the sample region and area with/without emergent vegetation, respectively. Elliptic regions distinguish samples by vegetation.(95% confidence intervals). Statistically significant environmental variables associated with changes in microbial community structure are plotted as vectors, where the length and direction indicate the degree of the correlation with the data.

Illumina sequencing of the 16S rRNA gene library from all 68 samples yielded a total of 6 905 068 reads. Following filtering, paired-end merging, and chimera removal, 1 578 781 merged reads remained, with between 5640 and 131 019 reads per sample (average 25 052). ASVs were rarefied to the minimum read count (5 640 ASVs) before multivariate analysis. Details regarding read filtering, forward and reverse sequencing quality, and the overall sequencing depth is summarized in Table S4. The most notable pattern of the overall microbial community is their separation according to geographic region (i.e. West vs. East, Fig. 2, PERMANOVA, *P* < 0.001). The distribution of microbial community composition was more variable among the western wetlands than among the eastern ones (Fig. 2). Additionally, alpha diversity indices showed lower microbial richness and diversity within the eastern wetlands (Fig. S1), indicating that regional differences were evident in both community variability and within-site diversity.

**Figure 3.**
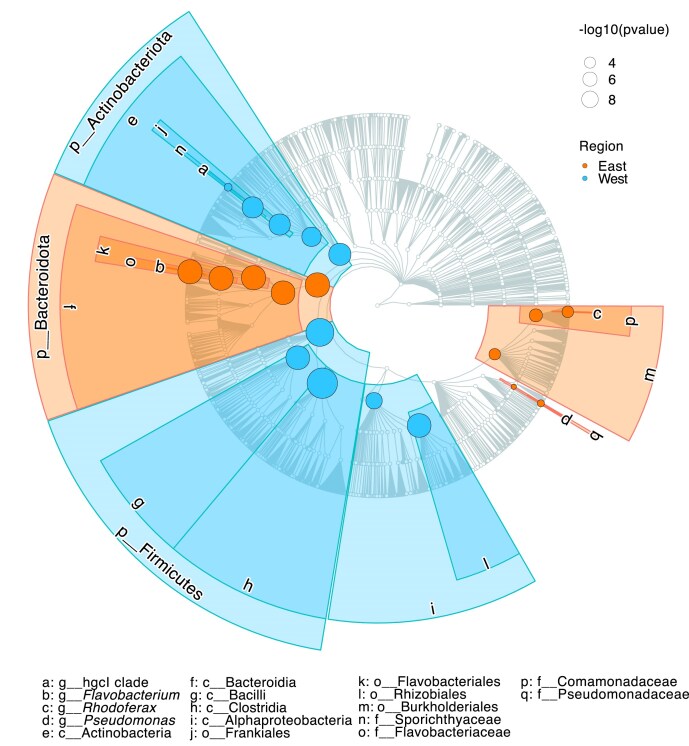
Phylogenetic tree plot with significant differences in relative abundances between western (Halland) and eastern (Mälardalen) wetland samples. The size of the dots represents the significance of the difference [−log_10_(*P*-value >4)] from Linear Discriminant Analysis. Identified taxa are labeled with letters and numbers on the plot and listed in the legend, with their taxonomic level indicated: class (c), order (o), family (f), and genus (g). Phylum-level assignments (p) are shown around the outer ring of the tree.

The apparent separation of microbial communities from the two regions was driven by a small number of key functional groups. Western (Halland) wetlands had significantly higher relative abundances of phyla with LDA scores >4, including Actinobacteriota, Firmicutes, and class Alphaproteobacteria (belonging to Pseudomonadota, formerly Proteobacteria) (Fig. 3). Using a relaxed LDA threshold (>3), additional phyla with higher relative abundances in the west included Acidobacteriota, Chloroflexi, Planctomycetota, Nitrospirota, Desulfobacterota, Verrucomicrobiota, and Gemmatimonadota. In contrast, eastern (Mälardalen) wetlands showed higher relative abundances of Bacteroidota and Pseudomonadota (Table S5).

At the genus level, community members contributing >1% of the total reads in at least one sample are presented in Fig. S2, whereas significant differences in relative genera abundance between the two regions are listed in Table S6. The top five taxa with significantly higher relative abundances in western wetlands compared to eastern sites were the *hgcI* clade, unclassified Sporichthyaceae, and *Mycobacterium* (all belonging to Actinobacteriota), as well as *Polynucleobacter* and Rhizobiales Incertae Sedis (both within Pseudomonadota). Conversely, taxa with significantly higher relative abundances in eastern wetlands included *Flavobacterium* and *Pedobacter* (both in Bacteroidota), along with *Polaromonas, Pseudomonas*, and *Rhodoferax* (all in Pseudomonadota) (Table S6).

**Figure 4.**
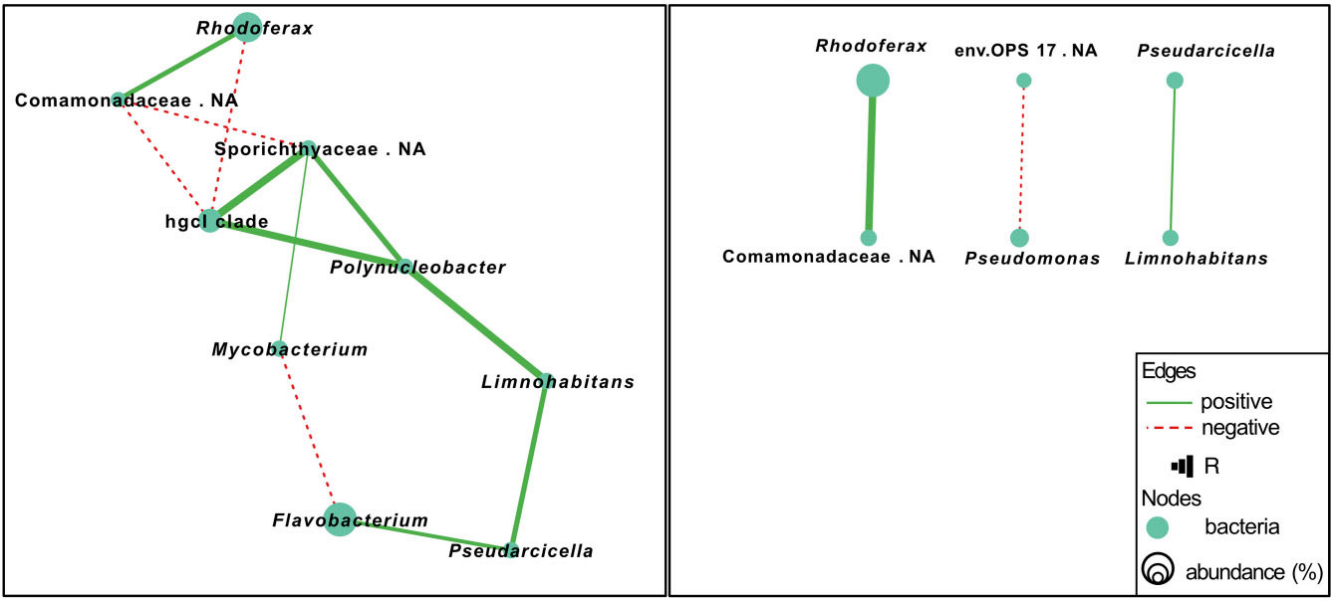
Co-occurrence network analysis based on the correlation of the relative abundance of amplicon sequence variant reads for genera or the closest classified taxonomic level (relative abundance >1%) for western (left) and eastern (right) wetland water samples. Each edge represents significant correlations between pairs of nodes (*p* < 0.05), where positive correlations are shown as solid lines and negative correlations as dotted lines. The thickness of the edge is proportional to the *ρ* value of the correlation (|*ρ*| > 0.5). Here the genus name could not be assigned to the sequences (NA), the closest classified taxonomic level is depicted.

To highlight the key microbial community that differed between the two regions, we conducted co-occurrence network analysis based on the spatial variation of relative abundance of 16S rRNA gene for samples between the wetland regions. Figure 4 presents significant correlations (*P* < 0.05, |*ρ*| > 0.5) in a microbial network constructed from genera with at least three nodes (each >1% relative abundance). Node properties such as degree, betweenness, closeness, and connectance centrality are provided in Table S7. Positive correlations between taxa suggest potential co-occurrence or shared environmental preferences, while negative correlations may reflect niche differentiation or competitive exclusion. For the western samples, there is a more complex network with many interconnected taxa while the eastern samples showed a simpler network with fewer connections and nodes.

One microbial cluster was observed in the western samples (Fig. 4, left), where the highly abundant *hgcI* clade (also known as Candidatus Nanopelagicales, phylum Actinobacteriota) showed strong positive correlations with *Polynucleobacter* (Pseudomonadota) and unclassified member of Sporichthyaceae (Actinobacteriota), as well as with *Mycobacterium* (Actinobacteriota). *Polynucleobacter* was further positively linked to *Limonohabitans* and *Pseudarcicella* (both Pseudomonadota) and *Flavobacterium* (Bacteroidota). *Flavobacterium* was negatively correlated with *Mycobacterium. Rhodoferax* and Comamonadaceae (Pseudomonadota) were positively associated with each other but negatively correlated with both *hgcI* clade and Sporichthyaceae.

For the eastern samples, no large microbial cluster was observed but a few isolated positive correlations between *Rhodoferax* and Comamonadaceae (both belonging to Pseudomonadota), *Pseudarcicella* and *Limonohabitans*, while there was a negative correlation between *Pseudomonas* and uncultured eubacterium env.OPS 17 (Fig. 4, right). The recurrence of positive correlations between *Pseudarcicella* and *Limonohabitans, Rhodoferax* and Comamonadaceae were independently observed in both regions, suggesting their universal symbiotic relationships in these constructed wetlands.

### Regional differences in methanogenesis and methanotrophic community

Although there were no significant differences between regions in measured CH_4_ concentrations in the water column (Table S3), the composition of methanogenic and methanotrophic communities varied notably.

**Figure 5.**
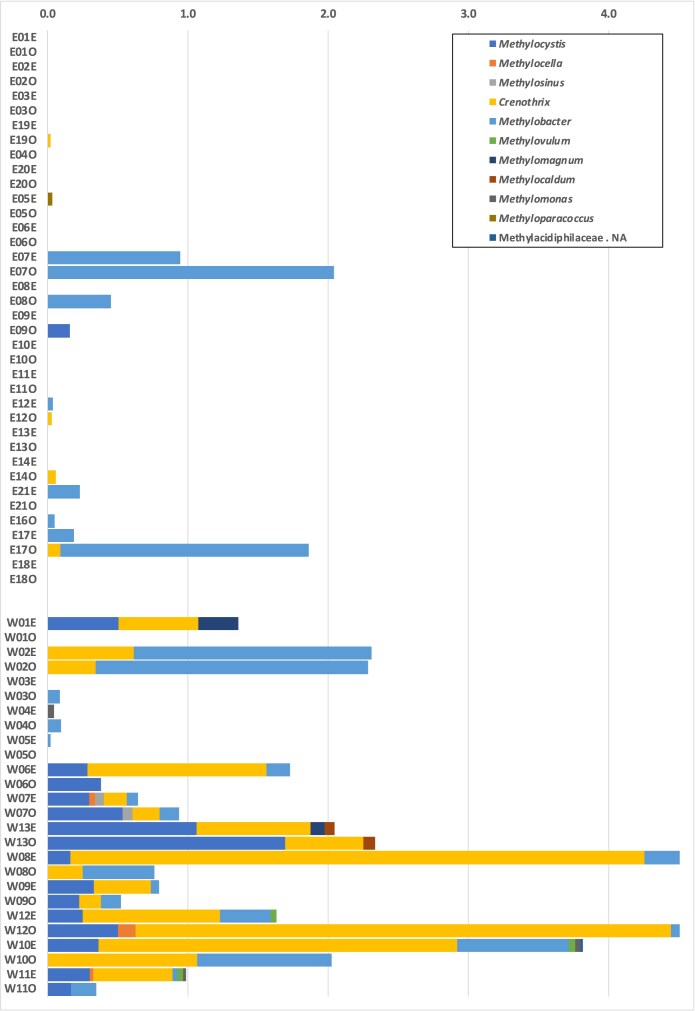
Methanotrophic community composition in constructed wetland (CW) samples. Samples are named according to a standardized code: the first letter indicates the region (“W” = west, “E” = east), the number corresponds to the sampling order (Table S1), and the final letter denotes vegetation status (“E” = with Emergent vegetation; “O” = Open water). The *x*-axis represents the relative abundance (%) of methanotrophs within the overall microbial community.

The genus *Methanocorpusculum* was the only detected methanogen in the water samples and appeared exclusively in the western samples, where it comprised up to 0.4% of the overall microbial community (Table S6). As for methanotrophs, the known type II methanotrophic genera *Crenothrix, Methylocystis*, and *Methylocella* were predominantly detected in the western samples, with average relative abundances of 0.7%, 0.3%, and <0.1%, respectively (Fig. 5). Among the type I methanotrophs, *Methylobacter* was the dominant genus, with an average relative abundance of 0.3% in the western samples. In contrast, these methanogens and methanotrophs were either absent or detected at much lower levels in the eastern water samples, except for samples from two wetlands where *Methylobacter* was dominant (Fig. 5).

### Emergent vegetation effects on the microbial community in water samples

There was no clear overall separation of microbial community distribution for samples from open water and with emergent vegetation areas in either region (Fig. 2, PERMANOVA, *P* > 0.05). However, alpha diversity analysis showed that microbial diversity and evenness, measured by Simpson and Shannon indices, were significantly higher in vegetated areas (*P* < 0.05). In contrast, ASV richness, assessed by both observed counts and the Chao1 index, did not differ significantly between the two habitat types (Fig. S3).

**Figure 6.**
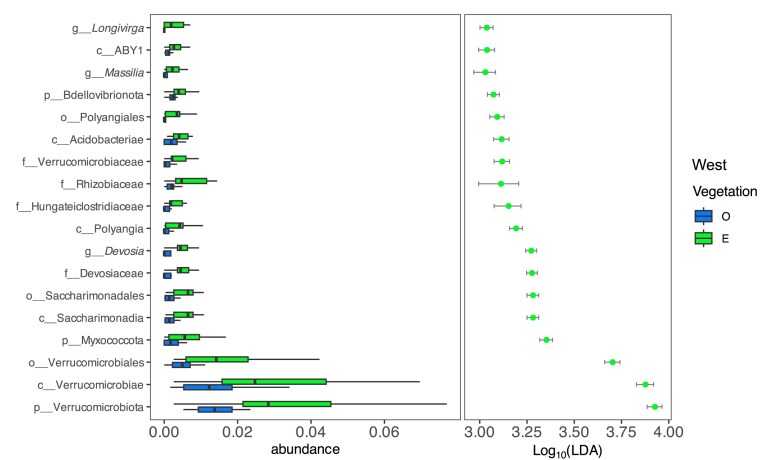
ASVs with significant differences in relative abundances between open water (O) and emergent vegetation (E) areas in Western wetlands. Relative abundances in the left panel and the confident interval of effect size presented as Linear Discriminant Analysis score in the right panel. Taxa were considered significantly different based on Kruskal–Wallis *P* < 0.05, followed by pairwise Wilcoxon tests (*P* < 0.05), and an LDA score threshold > 3.0. Taxa on the *y*-axis are ordered by increasing LDA score. The closest classified taxonomic level is depicted: g (genus), f (family), o (order), c (class), p (phylum).

To minimize possible confounding effects of sample region that may obscure the subtle difference of emergent vegetation vs. open water, we conducted the latter microbial community analysis separately for the western and eastern wetland regions. In eastern wetlands, no significant specific microbial differences were found between open water and emergent vegetation areas (according to LDA). Similarly, no difference was seen regarding alpha diversity index (Fig. S4, upper panels). For western wetlands, both richness and diversity index are clearly higher in vegetation areas (Fig. S4, lower panels). This enrichment was driven by several taxa that showed significantly higher relative abundance in emergent vegetation areas, including members of Verrucomicrobiota (e.g. Verrucomicrobiaceae, Verrucomicrobiales, Verrucomicrobiae), Acidobacteriota (e.g. class Acidobacteriae), Pseudomonadota (e.g. Devosiaceae, *Devosia*, Rhizobiaceae, *Massilia*), Bdellovibrionota, and Firmicutes (e.g. Hungateiclostridiaceae). Additional enriched groups included the genus *Longivirga*, order Saccharimonadales, and phylum Myxococcota (Fig. 6).

## Discussion

### Regional segregation of microbial communities

The clear separation in microbial community structure between western and eastern wetlands likely reflects strong biogeographic and environmental filtering. Western wetlands harbored more diverse microbial assemblages, including phyla such as Actinobacteriota and Firmicutes, which have members commonly associated with the degradation of complex organic matter (Bueno de Mesquita et al. 2024). In contrast, eastern wetlands showed higher relative abundances of Bacteroidia, which include taxa often linked to the processing of labile carbon compounds (Elovaara et al. 2021). Although CH_4_ concentrations did not significantly differ between regions, the contrasting microbial composition suggests different methane-cycling potentials. For instance, the higher relative abundance of heterotrophic decomposers in the east, coupled with elevated TOC and TP, may support methanogenesis under ice-covered conditions. In contrast, the microbial diversity and redox-sensitive taxa in the west suggest more complex carbon turnover networks that could favor methane oxidation, especially in oxygenated microzones (Hernandez et al. 2015). While ice cover may to some extents have contributed to these apparent differences (e.g. most eastern wetlands were ice-covered and western wetlands were not), ice-free wetlands in the east did not emerge as outliers. This suggests that ice cover alone cannot explain the regional differences and that other environmental and geographic factors (e.g. soil type, agricultural intensity, and wetland age) are likely causing the observed differences in resident microbial communities.

### Regional differences in methanogenic and methanotrophic communities

Methanogens were sparsely represented in both regions, reflecting the oxygenated nature of surface waters during winter, even with the ice cover present in the eastern wetlands. Notably, *Methanocorpusculum* was the only methanogen detected and was only found in western wetlands. This genus, a member of Methanomicrobiales, which has been previously identified as dominant in boreal freshwater systems (Bravo Andrea et al. 2018), and is known for producing methane under low-energy conditions, often utilizing hydrogen and CO₂ as substrates (Gilmore et al. 2017). The absence of common methanogenic archaea such as Methanobacteriales and Methanosarcinales suggests that methanogens are either extremely low in abundance in the winter water column or restricted to deeper sediment layers not well represented in surface water samples.

In contrast, methanotrophs were widely distributed across both regions. However, their diversity and relative abundance varied significantly between regions. Western wetlands had a higher overall relative abundance and diversity of methanotrophs, particularly Type II methanotrophs (*Methylocystis* and *Methylocella*), as well as the filamentous Type I methanotroph *Crenothrix* (Stoecker et al. 2006). Type II methanotrophs are known for their facultative lifestyles and association with vegetated, nitrogen-limited environments, where they can persist under fluctuating oxygen conditions (Dedysh Svetlana et al. 2005, Ho et al. 2016). Their predominance in the west may reflect such habitat conditions, including higher plant-mediated oxygenation and lower ammonium concentrations. In contrast, eastern wetlands were dominated by Type I methanotrophs and in particular *Methylobacter*, which was the most abundant methanotroph at some sites. *Methylobacter* species, such as *M. tundripaludum*, are well-known cold-adapted obligate methanotrophs commonly found in Arctic and boreal soils and sediments (Wang et al. 2024). They thrive in environments with high oxygen and methane availability and are especially active at low temperatures (Wartiainen et al. 2006). Genomic and physiological studies have revealed that they rely on the RuMP pathway for carbon assimilation, lack the serine cycle, and do not utilize multi-carbon substrates, indicating a strong dependence on methane and oxygen (Svenning Mette et al. 2011). Their prevalence in the east may therefore reflect a combination of low winter temperatures, surface water oxygenation, elevated phosphorus (P) concentrations. These characteristics highlight the specific physiological requirements of *Methylobacter*. Its dominance in the eastern wetlands, despite similar CH_4_ concentrations across regions, suggests that environmental filtering plays a stronger role in shaping winter methanotroph communities. In particular, nutrient status may be a key driver.

### Regional differences in key taxonomic group and the influence on methane cycling

Beyond methane-cycling microbes, the two regions differed in other key taxonomic groups relevant to carbon and nutrient transformations. Western sites, with higher relative abundances of Actinobacteriota (e.g. Sporichthyaceae *hgcI* clade, also called Candidatus Nanopelagicales) and Acidobacteriota, may support bacteria involved in degrading complex organic matter in soils and waters. In particular, Acidobacteriaceae, which encode diverse glycoside hydrolases and peptidases, supporting slow but sustained decomposition of recalcitrant organic matter under cold conditions (Gonçalves et al. 2024). Members of Firmicutes, especially class Bacilli and Clostridia, were also more abundant in the west. These results align with recent findings in constructed wetlands (Hartman Wyatt et al. 2024), where Firmicutes, particularly class Bacilli and Clostridia, were associated with elevated methane fluxes. Their study further highlighted how vegetation and seasonal dynamics shape microbial communities relevant to methane cycling. Although our study focuses exclusively on winter, the prevalence of Firmicutes in western wetlands may similarly reflect zones of active organic matter turnover and methane processing, even under ice-covered, low-light conditions. Eastern sites had higher relative abundances of Bacteroidota (*Flavobacterium*) and Pseudomonadota (*Pseudomonas*), which are also versatile aerobic heterotrophs that break down polymers and detritus (Jiménez et al. 2014, Ma et al. 2020). The contrasting nutrient profiles, higher TOC and phosphorus in the east vs. higher total nitrogen in the west, may influence microbial carbon processing. While functional roles cannot be fully resolved from taxonomy alone, these patterns are consistent with a shift toward more labile organic matter degradation in the east and potentially more complex or recalcitrant substrate processing in the west.

Sulfate-reducing bacteria were more abundant in western wetlands, with Desulfobacterota, a phylum of obligate anaerobic sulfate reducers (Hao et al. 2023), showing significantly higher relative abundance (LDA > 3). This may reflect more prevalent anoxic, sulfate-rich microenvironments in the west. Historically, sulfur deposition has been higher along Sweden's west coast (Moldan et al. 2013), which, while not directly indicating porewater sulfate levels, suggests greater regional sulfur availability. These conditions could give sulfate reducers a competitive advantage over methanogens for organic substrates (Lovley 2017), potentially contributing to the relatively lower methane concentrations observed in the west. In contrast, the east’s lower relative abundance of sulfate reducers may reflect reduced sulfate availability or environmental conditions that favor methanogenesis.

Microbial communities also differed in their nitrogen-transforming potential. Western wetlands had a higher relative abundance of Nitrospirota, a phylum primarily composed of nitrite-oxidizing bacteria such as *Nitrospira*, suggesting more active nitrification. This aligns with higher dissolved oxygen levels and lower ammonium concentrations observed in the west, as nitrification consumes oxygen and typically reduces ammonium (Daims et al. 2015). These patterns also correspond to higher total nitrogen concentrations in the west and the fact that many of these wetlands were constructed to enhance nitrogen removal. In contrast, eastern wetlands had fewer Nitrospirota and significantly higher ammonium concentrations, suggesting reduced nitrification capacity. Oxygen levels were significantly higher in the west, consistent with thinner or absent ice cover, while the lower concentrations in the east likely reflect more persistent ice and may influence redox-sensitive processes. These conditions could suppress nitrification and instead favor anaerobic nitrogen turnover. Supporting this, we observed higher relative abundances of facultative anaerobes such as *Pseudomonas* and *Rhodoferax*, which are capable of nitrate and iron reduction (Finneran et al. 2003, Wang et al. 2014). While denitrifier activity was not directly measured, the dominance of these taxa suggests that denitrification and related anaerobic pathways may play a more prominent role in nitrogen cycling within eastern wetlands during winter.

### Network analysis and microbial interconnectivity

The network analysis provide additional insights into microbial community interactions and their functional roles in the constructed wetland ecosystems. The more complex microbial network seen in western wetlands implies strong microbial interconnectivity, potentially contributing to more stable carbon and nutrient cycling processes. In contrast, the simpler network observed in eastern wetlands indicates a less interconnected microbial community, possibly reflecting lower microbial diversity and functional redundancy (Banerjee et al. 2016, Liu et al. 2024).

One of the key findings from the network analysis was the presence of a distinct bacterial cluster in western wetlands, primarily composed of the *hgcI* clade (Actinobacteriota), *Polynucleobacter* (Pseudomonadota), and *Sporichthyaceae* (Actinomycetota). The *hgcI* clade, identified as a core node in the western microbial network, likely contributes to winter microbial stability by transforming humic-derived dissolved organic matter (Zufiaurre et al. 2022). Through this initial cleavage of complex DOM, *hgcI* may indirectly influence the pool of substrates available for downstream methanogenesis. Recent genomic studies have revealed that these organisms are not only ultramicrobacteria with some of the smallest genomes among free-living microbes, but also exhibit auxotrophies that may create metabolic interdependencies within the community (Neuenschwander et al. 2018). This metabolic specialization and genomic micro-diversification may explain their ecological success and core positioning in networks shaped by seasonal carbon fluxes. *Polynucleobacter*, a dominant bacterioplankton genus, efficiently utilizes labile dissolved organic carbon such as acetate and pyruvate which are known photooxidation products from humic substances. Their high relative abundance and substrate preferences suggest a central role in freshwater carbon cycling, particularly in utilizing photooxidation products of humic substances (Hahn et al. 2012), potentially modulating substrate pools relevant to methane dynamics. Recent studies have also proposed that some freshwater Pseudomonadota (formerly Proteobacteria), including members of the *Polynucleobacter* genus, may harbor pathways such as methylphosphonate degradation that could contribute to aerobic methane production under specific environmental conditions, although direct evidence remains limited and context-dependent (Perez-Coronel and Michael Beman 2022).

*Sporichthyaceae* further contribute to organic matter degradation and nitrogen cycling by utilizing complex organic substrates and nitrite, supporting both carbon turnover and microbial community resilience during winter (Tamura 2014). This cluster was further linked to *Pseudarcicella* and *Flavobacterium* (Bacteroidota) through *Limonohabitans* (Pseudomonadota). *Pseudarcicella* and *Flavobacterium* are key players in the breakdown of complex organic matter, including algal exudates and plant-derived polymers, which are both processes that can influence methane production by modulating the availability of substrates for methanogenesis (Guo et al. 2021). The positive correlation between *Rhodoferax* and *Comamonadaceae* suggests potential functional roles in carbon turnover, particularly in oxygen-limited microenvironments where *Rhodoferax* can facilitate denitrification and iron reduction, processes that indirectly impact methane emissions (Jin et al. 2020). In contrast, the eastern wetland microbial network was less structured, showing only a few correlations. The positive relationships between *Pseudarcicella* and *Limonohabitans*, as well as *Rhodoferax* and *Comamonadaceae*, indicate key microbial interactions supporting essential biogeochemical processes persist in both regions. The negative correlation between *Pseudomonas* and an uncultured genus belonging to family env.OPS 17 may reflect niche differentiation, where generalists like *Pseudomonas* thrive in organic-rich, disturbed environments, while specialist taxa such as env.OPS 17 are adapted to stable environments with limited substrate diversity, relying on proteinaceous compounds rather than a broad spectrum of labile nutrients (Vieira et al. 2023).

These network patterns align with the broader differences observed in microbial communities. The highly interconnected community in the west, dominated by Actinobacteria and Firmicutes, suggests stronger microbial cooperation in carbon cycling and organic matter degradation, potentially enhancing methane oxidation or limiting substrates for methanogens during winter. While microbial communities in the west were more variable between sites, the aggregated network still showed higher complexity, suggesting that greater environmental heterogeneity across western wetlands may support both distinct local communities and more diverse inter-taxon interactions overall. In contrast, the simpler network observed in eastern wetlands, dominated by Bacteroidota, indicates a more selective community assembly, possibly shaped by winter ice cover, lower habitat heterogeneity, and more uniform redox conditions. Despite higher total organic carbon concentrations, eastern wetlands may offer less substrate diversity and reduced functional redundancy, potentially making key microbial processes such as decomposition, nitrification, and methane turnover more reliant on a narrower set of taxa and thus more sensitive to environmental change.

### Emergent vegetation influences on microbial communities

Our findings suggest that emergent vegetation exerted a limited influence on planktonic microbial community composition during winter, particularly when compared to strong regional effects. However, in western wetlands, areas with emergent vegetation supported higher alpha diversity, possibly due to accumulated organic matter and structurally complex microhabitats created by roots. This pattern was absent in the east, where emergent vegetation may play a less marked role in shaping microbial diversity under cold, ice-covered conditions.

In winter, reduced plant activity is likely to limit root exudation and coupled oxygen release. However, the presence of senescent plant material or submersed macrophytes may still provide a source of labile carbon, potentially supporting microbial activity under ice-covered conditions (Wang et al. 2023). Several taxa with increased relative abundance in sites with emergent vegetation may reflect functional adaptations that sustain biogeochemical cycling during dormancy: *Longivirga* (phylum Actinobacteriota) is a monotypic genus represented by *L. aurantiaca*, originally isolated from freshwater lake sediment (Qu et al. 2018). Although not previously linked to macrophytes, its presence in oligotrophic, oxygenated sediments suggests it may benefit from micro-oxic niches associated with emergent vegetation. *Devosia* (Pseudomonadota) is known for nitrogen fixation and plant-growth-promoting traits, including exopolysaccharide and phytohormone production (Rivas et al. 2003). Its presence suggests microbial nitrogen cycling may partially compensate for reduced plant uptake. *Massilia* (Pseudomonadota), also more abundant in zones with emergent vegetation, degrades cellulose and chitin and contributes to phosphate solubilization, supporting microbial activity near plant residues (Xiao et al. 2023). Acidobacteriae (Acidobacteriota), particularly subdivision 1, are known for their tolerance to fluctuating oxygen and nutrient levels, and some strains have been reported to produce indole-3-acetic acid and enhance iron availability (Kielak et al. 2016). Their potential involvement in ammonium cycling might partly explain the lower ammonium concentrations observed in the west. Saccharimonadales (Verrucomicrobiota), often found in nutrient-poor environments, may contribute to the slow degradation of complex plant material during winter dormancy (Moldovan et al. 2025), while Hungateiclostridiaceae (Firmicutes), a family of anaerobic fermenters, could remain active by utilizing residual organic matter from earlier seasons (Hao et al. 2024). Together, these taxa may reflect microbial strategies adapted to resource-scarce winter conditions, but further functional analysis would be needed to confirm their specific roles.

### Emergent-vegetation effects on methane cycling

Winter conditions also shape the relationship between vegetation and methane cycling. While CH_4_ concentrations did not differ significantly between emergent-vegetation and open water sites, the microbial communities in vegetated areas showed distinct shifts relative to unvegetated zones. These shifts included increased relative abundances of taxa potentially involved in fermentation and nitrogen cycling, which could indirectly influence methane production or oxidation. In warmer seasons, wetland plants transport oxygen to their roots, supporting methane-oxidizing bacteria (Ge et al. 2024), but in winter, reduced oxygen input may allow anoxic processes to dominate, potentially increasing methane accumulation in sediments (Laanbroek 2010).

The higher relative abundance of Hungateiclostridiaceae in vegetated areas may reflect locally enhanced organic inputs, such as senescent plant material, that sustain anaerobic degradation pathways under winter conditions. This could provide substrates for methanogens, influencing methane production under ice-covered conditions. Devosiaceae and *Devosia* were more abundant in emergent-vegetation areas, and their nitrogen-fixing potential may have indirectly supported methane oxidation by maintaining nitrogen availability for methanotrophs (Cui et al. 2022). Since nitrogen availability differed between regions, with eastern wetlands having higher ammonium concentrations and western wetlands higher TN, the influence of emergent vegetation on methane fluxes may be region-specific and dependent on nitrogen form.

### Broader implications and future directions

While our winter measurements revealed low but detectable methane concentrations in surface waters (mean: 0.015–0.017 mg l^−1^; maximum: 0.11 mg l^−1^), similar or even lower values have been reported in other systems during warmer periods. For instance, Johansson et al. (2004) measured methane emissions from a Swedish constructed wetland treating municipal wastewater and found seasonal fluxes ranging from −375 to 1739 mg m^−2^ d^−1^, with summer averages around 152–192 mg m^−2^ d^−1^ and much lower emissions in early spring and autumn. Methane fluxes during those shoulder seasons were reduced by factors of 10–50 compared to summer, and in some cases even showed net CH_4_ uptake (Johansson et al. 2004). Similarly, Dykes et al. (2025) observed fluxes ranging from 0.13 mg m^−2^ h^−1^ in autumn (below 10°C) to 20.5 mg m^−2^ h^−1^ in summer when water temperatures exceeded 22°C, again highlighting strong temperature dependence (Dykes et al. 2025). These patterns highlight that winter emissions, although lower, may still contribute substantially to annual GHG budgets, particularly in cold-climate regions where snow and ice can alter redox dynamics. Supporting this, Treat et al. (2018) reported that nongrowing-season CH_4_ emissions from natural wetlands contributed up to 29% of annual fluxes (Treat et al. 2018). However, similar seasonal quantification is lacking for constructed wetlands, and the influence of vegetation structure during winter is even less understood, despite its potential to modulate redox conditions and methane oxidation.

Beyond site-level observations, our results emphasize the need to evaluate the net greenhouse gas balance of constructed wetlands, weighing their nutrient retention capacity against CH_4_ and N₂O emissions. This trade-off is particularly relevant in agricultural landscapes where wetlands serve as nature-based nutrient mitigation tools. Recent studies show how design and stoichiometry influence these outcomes. Wu et al. (2025) reported that wetlands with influent COD: N ratios near 3 achieved effective nutrient removal with low CH_4_ and N₂O emissions (Wu et al. 2025). Jahangir et al. (2016) reviewed GHG emissions from different constructed wetland types and found that free water surface systems tend to emit more CH_4_ due to greater organic matter accumulation and persistent anoxic conditions, while subsurface flow systems are often more susceptible to N₂O emissions resulting from incomplete denitrification (Jahangir et al. 2016).

Our winter microbial data, particularly the distribution of methanotrophs, denitrifiers, and plant-associated taxa, contribute a cold-season perspective to understanding how emergent vegetation and microbial communities interact to shape greenhouse gas dynamics in constructed wetlands. The presence of redox-sensitive taxa suggests that wetland function persists under ice, though microbial pathways likely shift with oxygen availability and nutrient inputs. Importantly, by identifying microbial indicators such as methanotrophs and denitrifiers that respond to vegetation, nutrient levels, and seasonal conditions, our study lays the groundwork for integrating microbial data into monitoring and modeling frameworks. These indicators may serve as early signals of methane cycling or nitrogen transformation potential under winter conditions. Incorporating such microbial metrics into process-based models could improve predictions of CH_4_ and N₂O emissions across seasons and help guide adaptive wetland design. This integrated approach is essential to support both water quality and climate mitigation goals in constructed wetland management.

## Conclusion

This study highlights key regional and emergent vegetation-driven differences in microbial communities and methane cycling potential in constructed wetlands during winter. Western wetlands had higher microbial diversity and more interconnected networks, while eastern wetlands were simpler and more selective in composition. Vegetation-influenced microbial diversity is only seen in the West, with positive selection for known plant-associated microbes.

These findings support the hypothesis that emergent vegetation influences microbial diversity in cold climates, but no corresponding effect on water methane concentrations was observed. Still, this study provides valuable insights into microbial community structure and methane cycling in wetlands during winter, offering one of the few detailed examinations of microbial interactions under cold conditions. Integrating microbial network analysis and environmental data provides insight into how vegetation influences microbial diversity and methane oxidation, particularly under low temperatures. Certain limitations still remain. While network topology reflects potential ecological associations, co-occurrence does not confirm direct biological interactions. Functional assays are required to validate these relationships. Additionally, a thorough plant species survey, including potential winter-active submersed macrophytes, would help refine the evaluation of vegetation’s role in methane cycling. Addressing these knowledge gaps could inform future studies and help identify management actions, such as vegetation planning or sediment removal, that balance methane mitigation with other wetland functions in cold climates.

## Supplementary Material

fiaf086_Supplemental_Files
