# Supplementary material for: Winter microbial community structure and methane-cycling potential in constructed agricultural wetlands across regions and microhabitats
Source: FEMS Microbiol Ecol. 2025 Sep 3;101(9):fiaf086. doi: 10.1093/femsec/fiaf086 (PMC12421997; doi:10.1093/femsec/fiaf086)
Supplement: fiaf086_Supplemental_Files [file fiaf086_supplemental_files.zip › 20250828_Supplementary figures.pdf]

Supplementary figures

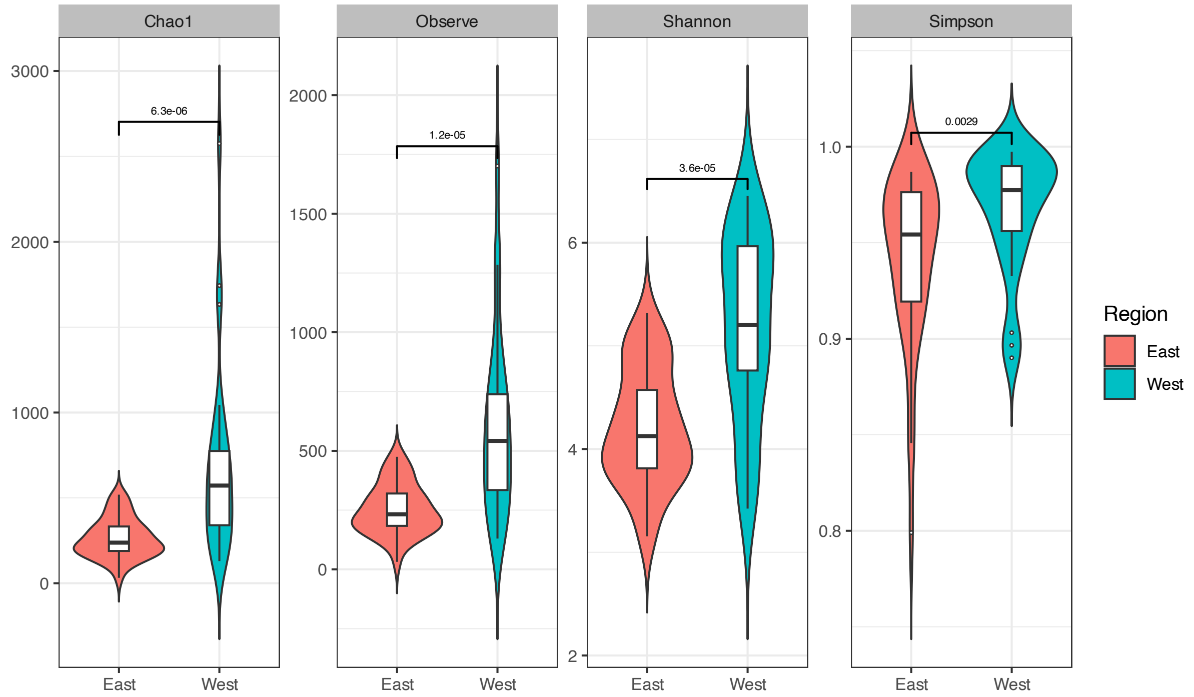

Figure S1. Violin plot representing rarefied counts of Amplicon Sequence Variants (ASVs) at a depth of 5,640 (the lowest counts across all samples) for alpha diversity analysis (Chao1: estimated ASV richness; Observe: observed ASVs richness; Simpson: amplicon sequence variants (ASVs) diversity, and Shannon: ASVs evenness). The significance (p-values) was presented between two groups of samples for each diversity indices.

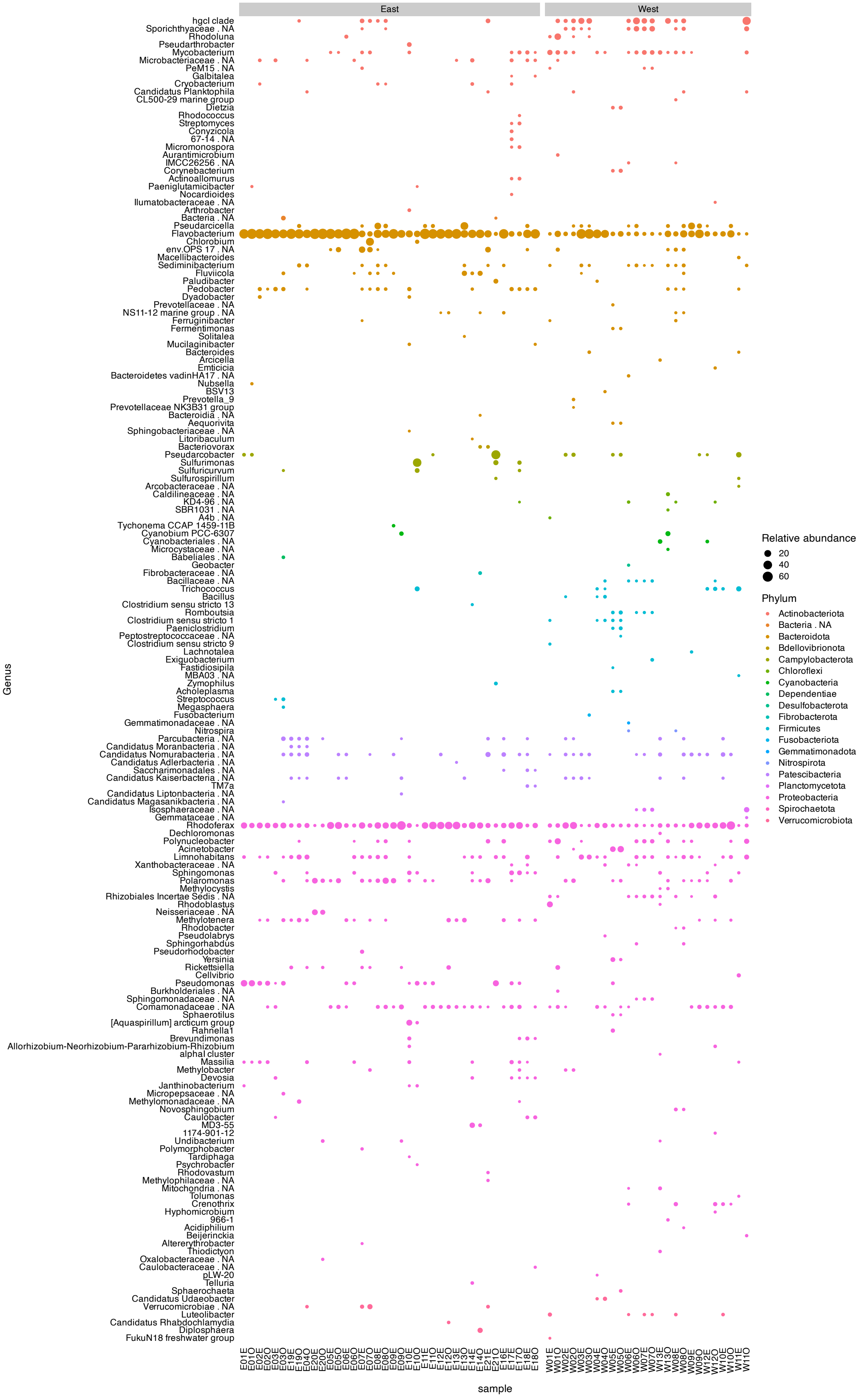
East
West
Genus
hgcI clade
Sporichthyaceae . NA
Rhodoluna
Pseudarthrobacter
Mycobacterium
Microbacteriaceae . NA
PeM15 . NA
Galbitalea
Cryobacterium
Candidatus Planktophila
CL500-29 marine group
Dietzia
Rhodococcus
Streptomyces
Conyzicola
67-14 . NA
Micromonospora
Aurantimicrobium
IMCC26256 . NA
Corynebacterium
Actinoallomurus
Paeniglutamicibacter
Nocardioides
Ilumatobacteraceae . NA
Arthrobacter
Bacteria . NA
Pseudarcicella
Flavobacterium
Chlorobium
env.OPS 17 . NA
Macellibacteroides
Sediminibacterium
Fluviicola
Paludibacter
Pedobacter
Dyadobacter
Prevotellaceae . NA
NS11-12 marine group . NA
Ferruginibacter
Fermentimonas
Solitalea
Mucilaginibacter
Bacteroides
Arcicella
Emticicia
Bacteroidetes vadinHA17 . NA
Nubsella
BSV13
Prevotella_9
Prevotellaceae NK3B31 group
Bacteroidia . NA
Aequorivita
Sphingobacteriaceae . NA
Litoribaculum
Bacteriovorax
Pseudarcobacter
Sulfurimonas
Sulfuricurvum
Sulfurospirillum
Arcobacteraceae . NA
Caldilineaceae . NA
KD4-96 . NA
SBR1031 . NA
A4b . NA
Tychonema CCAP 1459-11B
Cyanobium PCC-6307
Cyanobacteriales . NA
Microcystaceae . NA
Babeliales . NA
Geobacter
Fibrobacteraceae . NA
Bacillaceae . NA
Trichococcus
Bacillus
Clostridium sensu stricto 13
Romboutsia
Clostridium sensu stricto 1
Paeniclostridium
Peptostreptococcaceae . NA
Clostridium sensu stricto 9
Lachnotalea
Exiguobacterium
Fastidiosipila
MBA03 . NA
Zymophilus
Acholeplasma
Streptococcus
Megasphaera
Fusobacterium
Gemmatimonadaceae . NA
Nitrospira
Parcubacteria . NA
Candidatus Moranbacteria . NA
Candidatus Nomurabacteria . NA
Candidatus Adlerbacteria . NA
Saccharimonadales . NA
Candidatus Kaiserbacteria . NA
TM7a
Candidatus Liptonbacteria . NA
Candidatus Magasanikbacteria . NA
Isosphaeraceae . NA
Gemmataceae . NA
Rhodoferax
Dechloromonas
Polynucleobacter
Acinetobacter
Limnohabitans
Xanthobacteraceae . NA
Sphingomonas
Polaromonas
Methylocystis
Rhizobiales Incertae Sedis . NA
Rhodoblastus
Neisseriaceae . NA
Methylotenera
Rhodobacter
Pseudolabrys
Sphingorhabdus
Pseudorhodobacter
Yersinia
Rickettsiella
Cellvibrio
Pseudomonas
Burkholderiales . NA
Sphingomonadaceae . NA
Comamonadaceae . NA
Sphaerotilus
[Aquaspirillum] arcticum group
Rahnella1
Brevundimonas
Allorhizobium-Neorhizobium-Pararhizobium-Rhizobium
alphaI cluster
Massilia
Methylobacter
Devosia
Janthinobacterium
Micropepsaceae . NA
Methylomonadaceae . NA
Novosphingobium
Caulobacter
MD3-55
1174-901-12
Undibacterium
Polymorphobacter
Tardiphaga
Psychrobacter
Rhodovastum
Methylophilaceae . NA
Mitochondria . NA
Tolumonas
Crenothrix
Hyphomicrobium
966-1
Acidiphilium
Beijerinckia
Altererythrobacter
Thiodictyon
Oxalobacteraceae . NA
Caulobacteraceae . NA
pLW-20
Telluria
Sphaerochaeta
Candidatus Udaeobacter
Verrucomicrobiae . NA
Luteolibacter
Candidatus Rhabdochlamydia
Diplosphaera
FukuN18 freshwater group
E01E
E01O
E02E
E02O
E03E
E03O
E19E
E19O
E04O
E20E
E20O
E05E
E05O
E06E
E06O
E07E
E07O
E08E
E08O
E09E
E09O
E10E
E10O
E11E
E11O
E12E
E12O
E13E
E13O
E14E
E14O
E21E
E21O
E16E
E17E
E17O
E18E
E18O
W01E
W01O
W02E
W02O
W03E
W03O
W04E
W04O
W05E
W05O
W06E
W06O
W07E
W07O
W13E
W13O
W08E
W08O
W09E
W09O
W12E
W12O
W10E
W10O
W11E
W11O
sample
Relative abundance
20
40
60
Phylum
Actinobacteriota
Bacteria . NA
Bacteroidota
Bdellovibrionota
Campylobacterota
Chloroflexi
Cyanobacteria
Dependentiae
Desulfobacterota
Fibrobacterota
Firmicutes
Fusobacteriota
Gemmatimonadota
Nitrospirota
Patescibacteria
Planctomycetota
Proteobacteria
Spirochaetota
Verrucomicrobiota

Figure S2. Relative abundance of the bacterial community based on the average amplicon sequence variants (ASVs) reads of 16S rRNA genes from samples grouped according to the sample region. Taxonomy were list on genus level (left y-axis) and colored according to the phylum (right legend). Circle sizes represent the relative abundance of each identified genus. Here the genus name could not be assigned to the sequences (NA), the closest classified taxonomic level is depicted: class (C), order (O), and family (F). n=3. Relative abundance >1% in at least one sample is depicted.

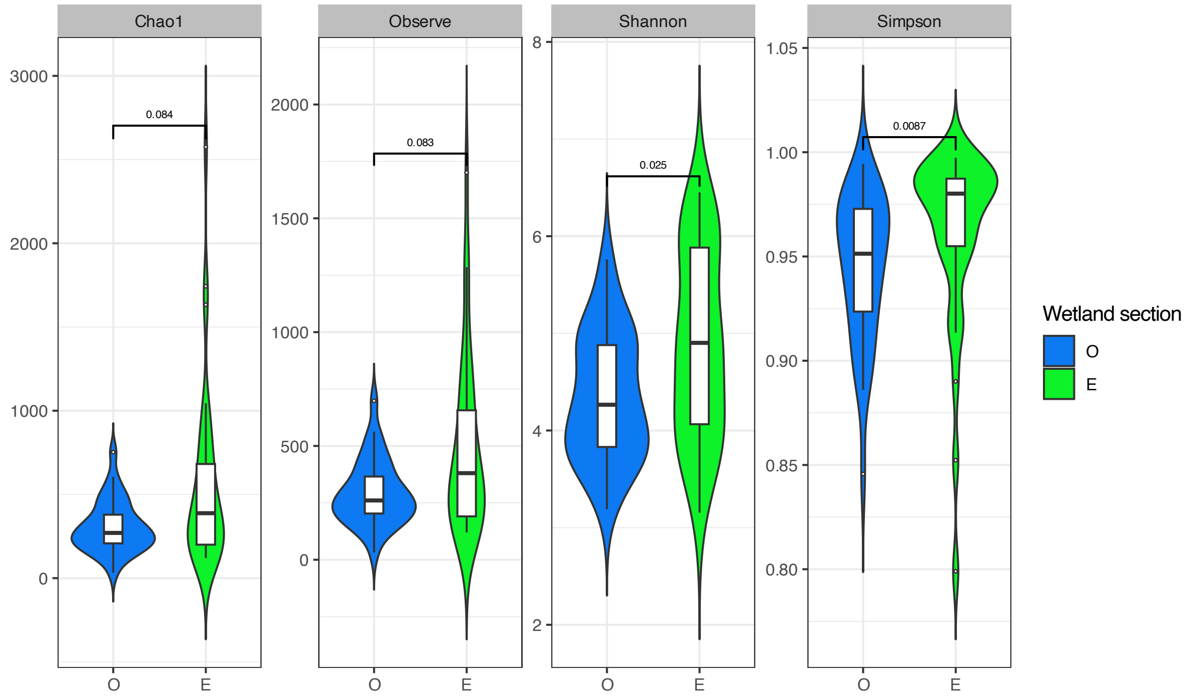

Figure S3. Violin plot representing rarefied counts of Amplicon Sequence Variants (ASVs) at a depth of 5,640 (the lowest counts across all samples) for alpha diversity analysis in areas with emergent vegetation (E) and open water (O)of all wetlands. (Chao1: estimated ASV richness; Observe: observed ASVs richness; Simpson: amplicon sequence variants (ASVs) diversity, and Shannon: ASVs evenness). The significance (p-values) was presented between two groups of samples for each diversity indices.

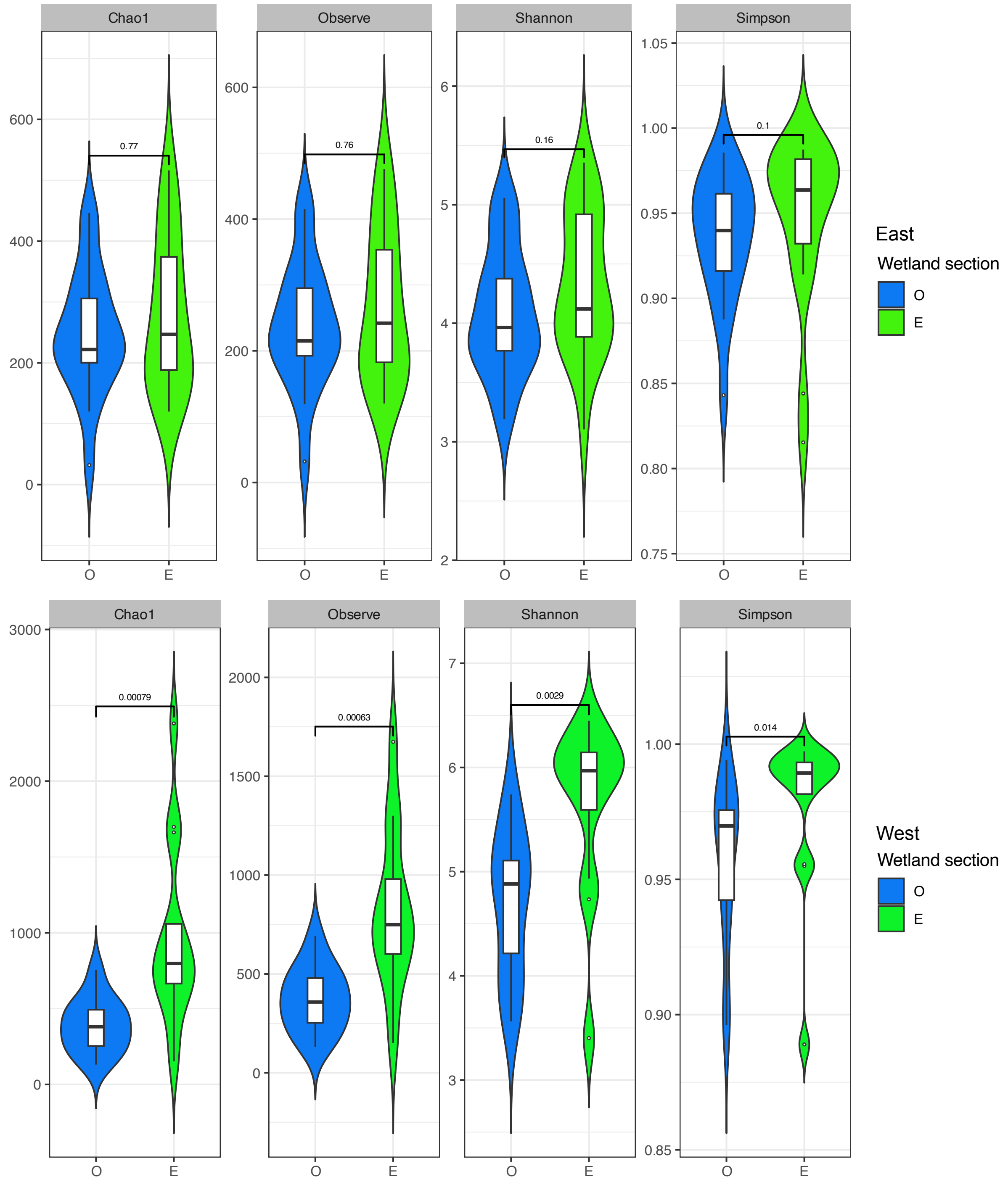

Figure S4. Violin plot representing rarefied counts of Amplicon Sequence Variants (ASVs) at a depth of 5,640 (the lowest counts across all samples) for alpha diversity analysis in areas with emergent vegetation (E) and open water (O) of eastern wetlands (upper panels) and western wetlands (lower panels). (Chao1: estimated ASV richness; Observe: observed ASVs richness; Simpson: amplicon sequence variants (ASVs) diversity, and Shannon: ASVs evenness). The significance (p-values) was presented between two groups of samples for each diversity indices.
